# Supplementary material for: Red blood cell-derived semaphorin 7A promotes thrombo-inflammation in myocardial ischemia-reperfusion injury through platelet GPIb
Source: Nat Commun. 2020 Mar 11;11:1315. doi: 10.1038/s41467-020-14958-x (PMC7066172; doi:10.1038/s41467-020-14958-x)
Supplement: Supplementary file 1 — Supplementary Information [file 41467_2020_14958_MOESM1_ESM.pdf]

<u>**Supplementary Information**</u>

Red Blood Cell derived Semaphorin 7A promotes thrombo-inflammation in myocardial ischemia-reperfusion injury through platelet GPIb

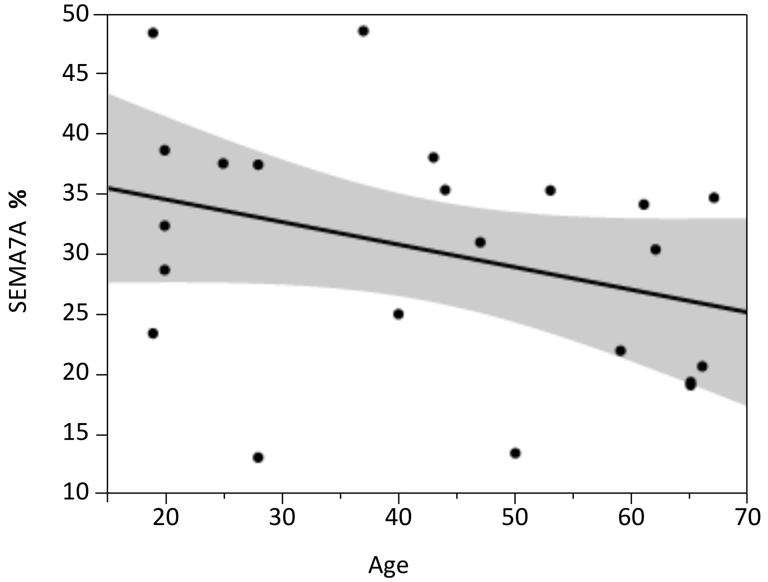

Supplementary Figure 1. Expression of SEMA7A (%) on human erythrocytes compared to age in years. Erythrocytes were analyzed in different age groups to determine the degree of SEMA7A expression on their surface. Comparison of SEMA7A % on human erythrocytes to years of donors by ANOVA showed no significant differences change over age p = 0.1194

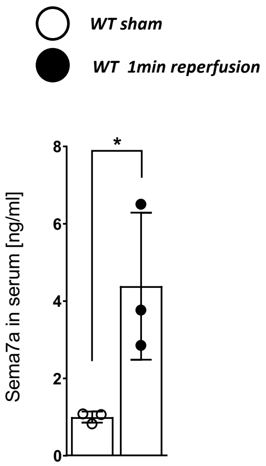

Supplementary Figure 2. Plasma values of Sema7a in WT mice undergoing myocardial ischemia and 1 min of reperfusion. WT mice were exposed to 60 minutes of ischemia. Samples were taken after 1 minute of reperfusion. Comparisons in this figure were analyzed by unpaired two-tailed Student's *t*-tests (data are mean±SD; n=3/ group; *p < 0.05 as indicated)

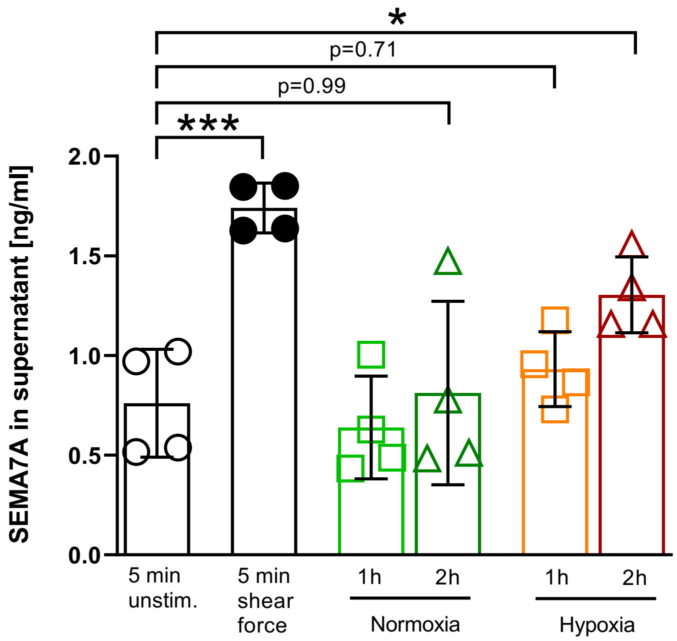

Supplementary Figure 3. SEMA7A cleavage from human erythrocytes. Human erythrocytes were exposed either to shear stress, hypoxia (2%O_2) or normoxia (21% O_2) for indicated time points and SEMA7A levels were determined in the supernatant. For comparisons in this figure we performed one-way analyses of variance followed by Dunnett's tests to group "5min unstimulated" (data are mean±SD; n=4/ group; *$p < 0.05$, ***$p < 0.01$ as indicated).

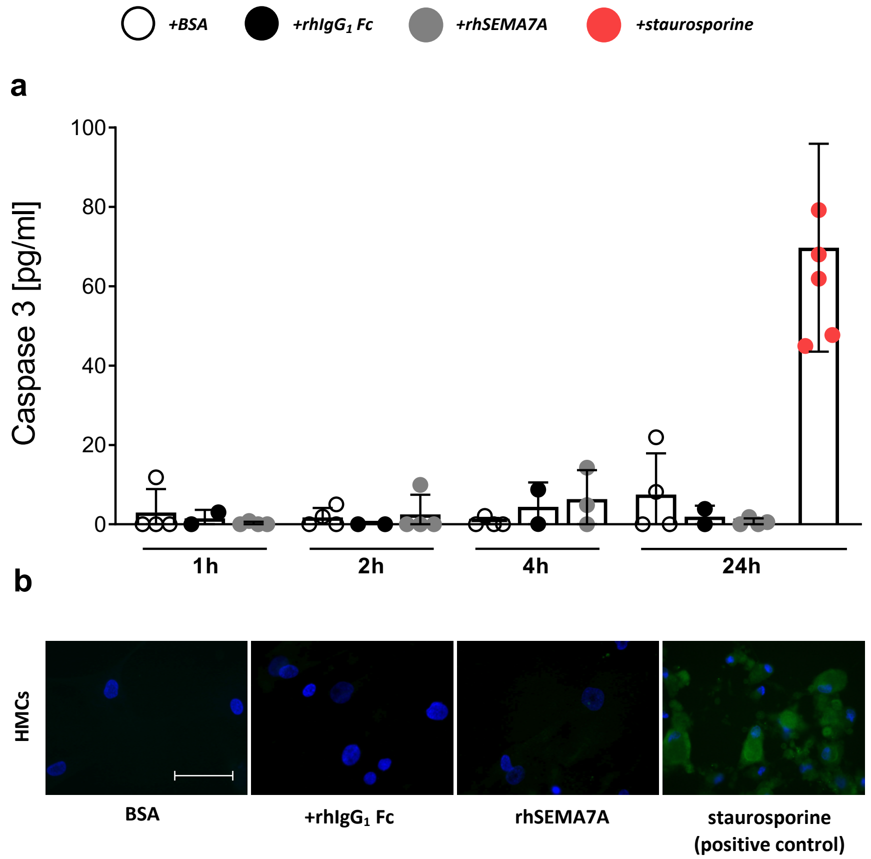

Supplementary Figure 4. SEMA7A does not induce caspase 3 in human myocardial cells (HMCs). a) HMCs were exposed to recombinant human SEMA7A (rhSEMA7A), appropriate Fc control (rhIgG$_1$ Fc), BSA only or staurosporine for the indicated times, and caspase 3 expression was measured by ELISA. **b)** HCMs were exposed to SEMA7A, Fc control or BSA only for 6 h and stained for caspase 3 (scale bar 100μm). Staurosporine treatment served as a positive control. Data are mean±SD; n=4;2;4;4;2;4;4;2;3;4;2;4;6, histology n=3.

a

b

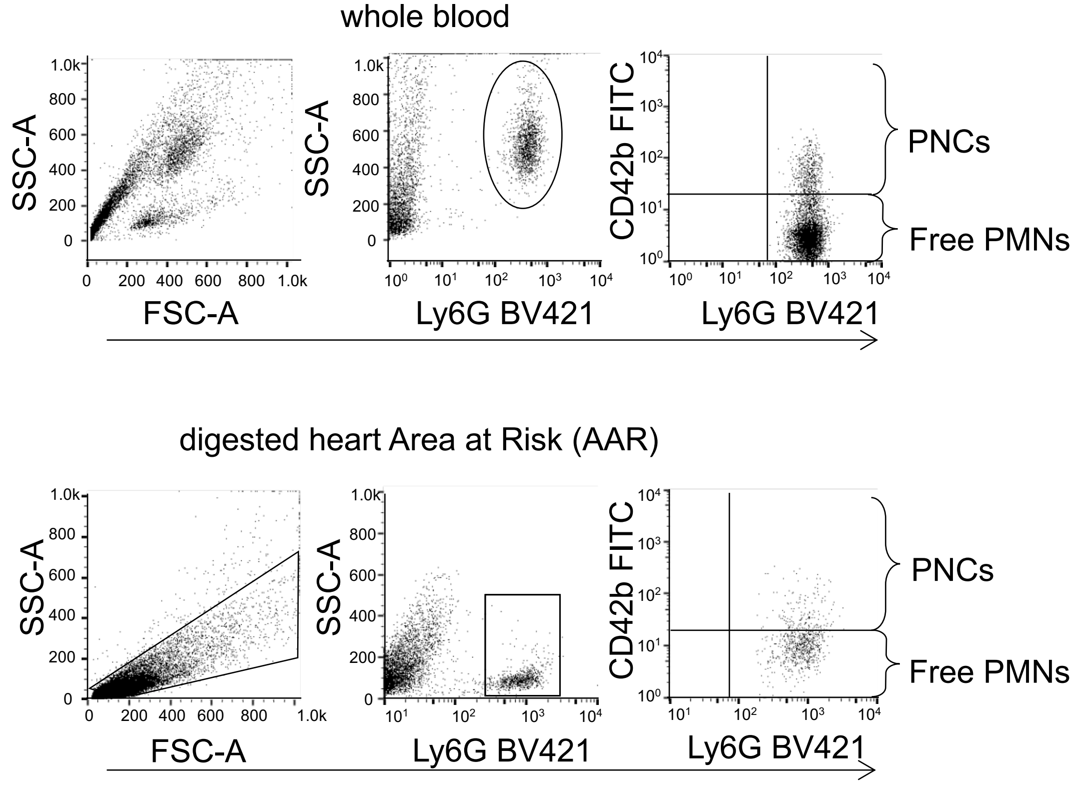

Supplementary Figure 5. Gating strategy for flow cytometry. Strategy to identify platelets, neutrophils and platelet-neutrophil complexes in the blood and area at risk of animals following myocardial ischemia reperfusion. **a)** Whole blood platelet -neutrophil aggregates (PNCs) were identified by flow cytometry. Red blood cells were lysed and whole blood samples fixed before flow cytometry acquisition. Whole-blood granulocyte population was focused by granularity (SSC) and expression of surface Ly6G (antibody labeled with BV421); platelet-neutrophil aggregates (PNCs) were recorded as Ly6G$^+$ granulocytes labeled positive foranti-platelet CD42b (antibody conjugated with FITC). This whole blood gating strategy was used to retrieve the data shown in Figures 2e, f, g, h; 3g, h, i, j; 6e, f, g, h and Supplementary Figures 7a, b; 9a, b; 13a, b **b**) After reperfusion, the cardiac area at risk was minced, digested and concentrated for staining with fluorescent antibodies. Tissue granulocytes were identified by their granularity (SSC) and expression of surface Ly6G (antibody labeled with BV421); myocardial platelet-neutrophil aggregates (PNCs) were recorded as Ly6G$^+$ granulocytes labeled positive with anti-platelet CD42b (antibody conjugated with FITC). This myocardial AAR gating strategy was used to retrieve the data shown in Figures 2i, j k, l; 3k, l, m, n; 6i, j, k, l and Supplementary Figures 7c, d; 9c, d; 13c, d.

For each sample, ≥ 10^4 events were acquired on a FACSCanto II, and data were analyzed with FlowJo 10.5. while conducting studies concentrated on platelet function and aggregate formation a platelet threshold has to be set to discriminate background auto-fluorescence from true platelet events [2,3]. Also in those studies we could identify the presence of platelet-neutrophil aggregates (PNCs) though in minimal concentration due to the discrepancy between platelets and PNC numbers. In summary, only events in whole blood samples or myocardial heart tissues carrying SSC/Ly6G/CD42b positive signal were further examined for their surface expression of CD62P or JON/A.

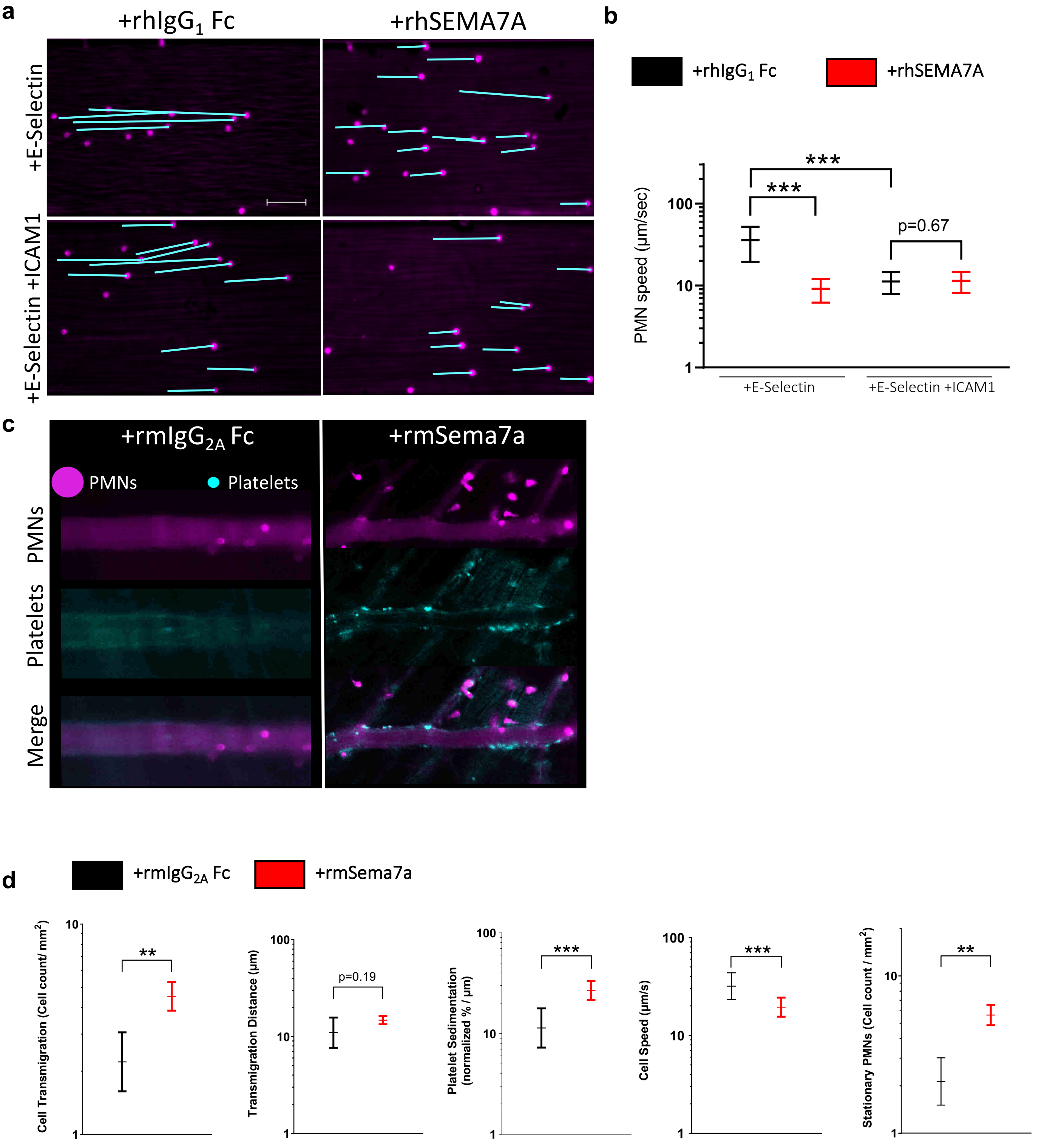

Supplementary Figure 6. SEMA7A influences neutrophil migration under flow. Whole blood neutrophils treated with rhSEMA7A and marked with rhodamine adhere to coated glass capillaries with E-selectin or E-selectin with ICAM1. **a)** Representative frame (scale bar 100µm) of neutrophil (magenta) tracks (cyan) on coated capillaries. Flow chamber experiments were performed with samples of 4 independent healthy donors and at least 150 cells were tracked in each experimental group. **b)** Neutrophil speed (µm / sec) on coated capillaries under the influence of rhSEMA7A (n=12;15;15;20) **c)** Representative images of neutrophils (magenta) and platelets (cyan) imaged under flow on living cremaster tissues before and after 15min of i.v. inoculation of rmSema7a. Intra vital microscopy experiments were performed on n=4 mice with n=10 videos per condition from rmSema7a groups acquired 15minutes after baseline control. **d)** From the intra-vital-microscopy videos acquired, neutrophil speed (µm / sec n=22;34) was calculated from tracked cells; stationary neutrophils count (cell count / mm^2 n=9;39) platelet sedimentation on the vascular wall was measured (normalized MFI % / mm n=18;29); transmigrated cells from the vasculature after 15min exposure to rmSema7a were count (cell count / mm^2 n=4;35); the distance of transmigrated cells measured in µm (9;177). Intra vital microscopy experiments were performed on n=5 mice with videos of 10 seconds (n=10 videos per condition with 20seconds of interval) from rmSema7a groups acquired 15minutes after baseline control. Data is represented as geometric mean with CI with P values designated as *p<0.05, **p<0.01, ***p<0.001.

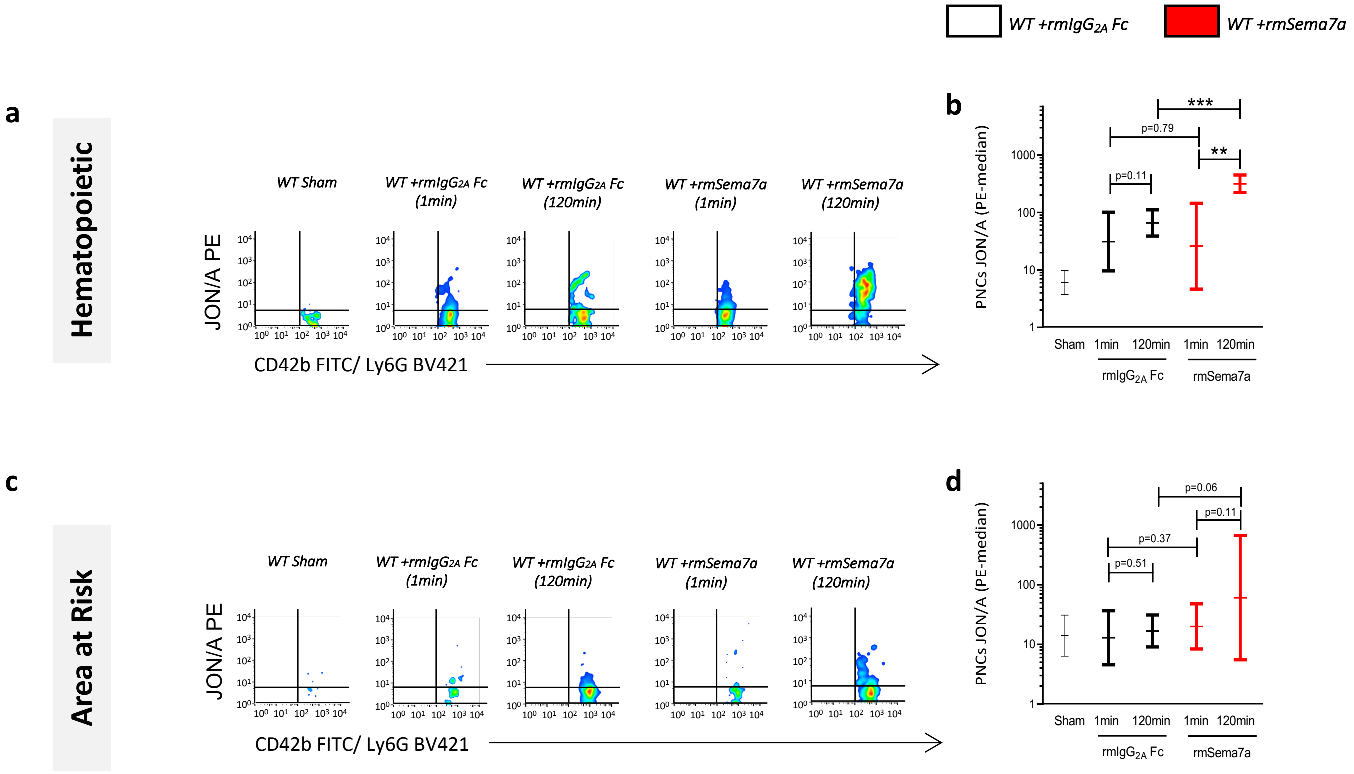

Supplementary Figure 7. Injection of Sema7a results in increased αIIbβ3 activation of PNCs. Animals were injected with either recombinant semaphorin 7a (rmSema7a) or Fc control (rmIgG$_{2A}$ Fc) and then subjected to 1 h of ischemia. Blood and myocardial tissue sections (AAR) were taken after 1 min or 120 min reperfusion. **a, c**) Representative flow-cytometric plots of PNCs in the blood and tissue of sham, rmSema7a Fc or IgG Fc control injected mice, showing αIIbβ3 (JON/A) activation. **b, d**) Systematic evaluation of flow-cytometric mean fluorescence intensities (MFI) of αIIbβ3 (JON/A) on PNCs (Hematopoietic n=7;4;4;4;4/ Area at Risk n=7;4;4;4;3) of rmSema7a-injected, IgG Fc control-injected or Sham mice. For this figure we used log transformation of data to conform normality. For log-transformed data, unpaired two-tailed Student's *t*-tests were performed on the log values and results are displayed as geometric means and their 95% confidence intervals. (**$p < 0.01$ and ***$p < 0.001$).

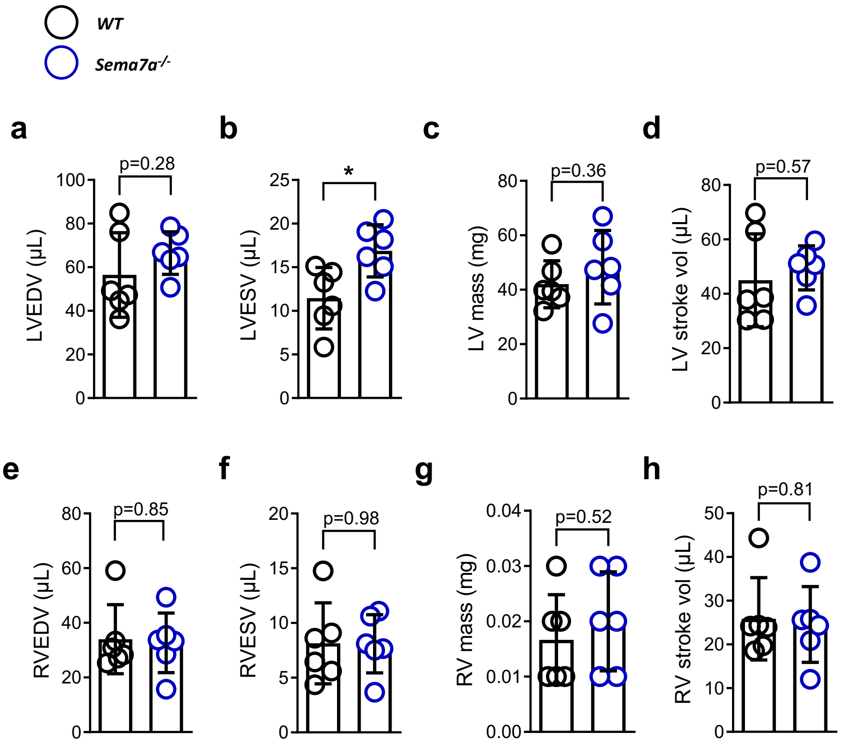

Supplementary Figure 8. ***Sema7a***$^{-/-}$ **animals do not show altered cardiac performance compared to WT littermates.** Short-axis cardiac MRI images were acquired on a 70/39 7T BioSpec scanner using the IntraGateFLASH measuring method with the following parameters: echo time = 2.112 ms, 100 repetitions of 74.352 ms, 128-acquisition square matrix, spatial resolution of 176 µm. Ten axial slices of 1 mm thickness and 10 cardiac frames for reconstruction were taken to cover RV and LV. **a)** Assessment of left ventricular end-diastolic volume (LVEDV), **b)** left ventricular end-systolic volume (LVESV), **c)** left ventricular mass (LV mass), and **d)** left ventricular stroke volume (LV stroke). **e)** Assessment of right ventricular end-diastolic volume (RVEDV), **f)** right ventricular end-systolic volume (RVESV), **g)** right ventricular mass (RV mass), and **h)** right ventricular stroke volume (RV stroke). Comparisons were analyzed by unpaired two-tailed Student's *t*-tests (data are mean±SD; n=6/ group, *p < 0.05 as indicated).

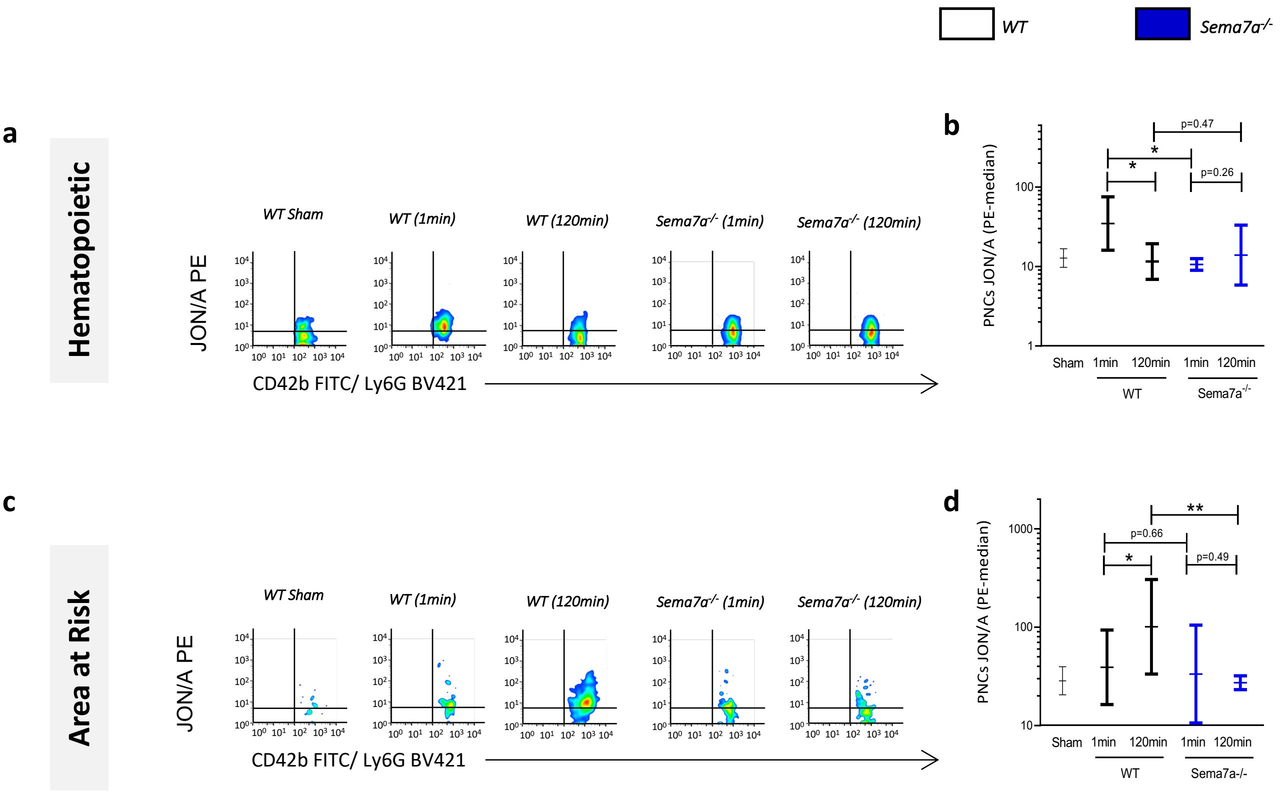

Supplementary Figure 9. *Sema7a$^{-/-}$* animals show reduced signs of αIIbb3 (JON/A) activation on PNCs. *Sema7a$^{-/-}$* and littermate controls were exposed to 1 h ischemia. Blood and myocardial tissue samples (AAR) were taken after 1 minute or 120 minutes of reperfusion. **a, c)** Representative flow-cytometric plots of PNCs in the blood (n=4;5;3;3;3) and tissue (n=4;3;3;3;3) of sham animals, *Sema7a$^{-/-}$* and littermate controls showing activated *α*IIbβ3 (JON/A) activation on PNCs. **b, d)** Systematic evaluation of flow-cytometric mean fluorescence intensities of *α*IIbβ3 (JON/A) on PNCs of *Sema7a$^{-/-}$* and littermate controls. For this figure we used log transformation of data to confirm normality. For log-transformed data, unpaired two-tailed Student's *t*-tests were performed on the log values and results are displayed as geometric means and their 95% confidence intervals. (*p <0.05 and **p < 0.01).

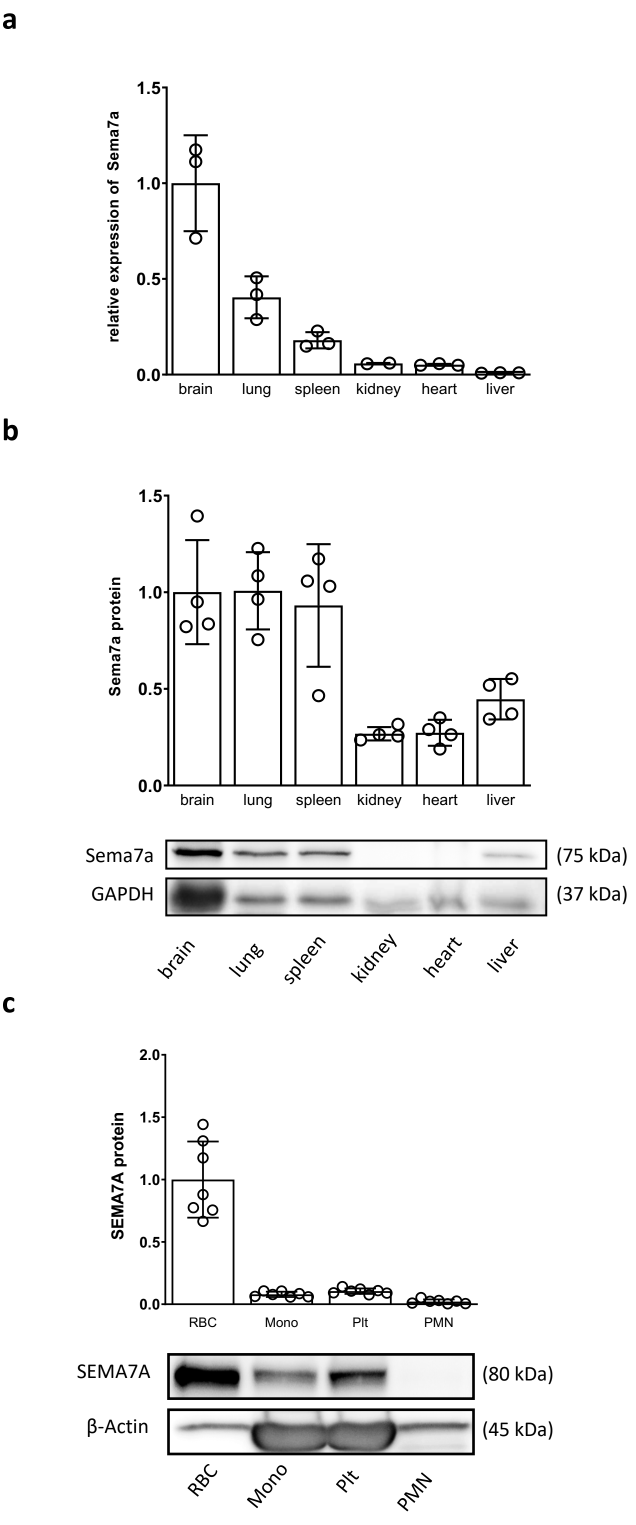

Supplementary Figure 10. Sema7a expression in tissue of mice and hematopoietic human cells. a) Relative Sema7a mRNA expression in murine tissues compared to brain (n=3;3;3;2;3;3). **b)** Densitometry of Sema7a protein expression in murine tissues with one representative western blot illustration (n=4/ group). **c)** Densitometry of Sema7a protein expression on human hematopoietic cells with one representative Western blot illustration (n=7/ group). RBC=Red Blood cells, Mono=monocytes, Plt=platelets and PMNs=polymorphonuclear cells. (all data are mean±SD).

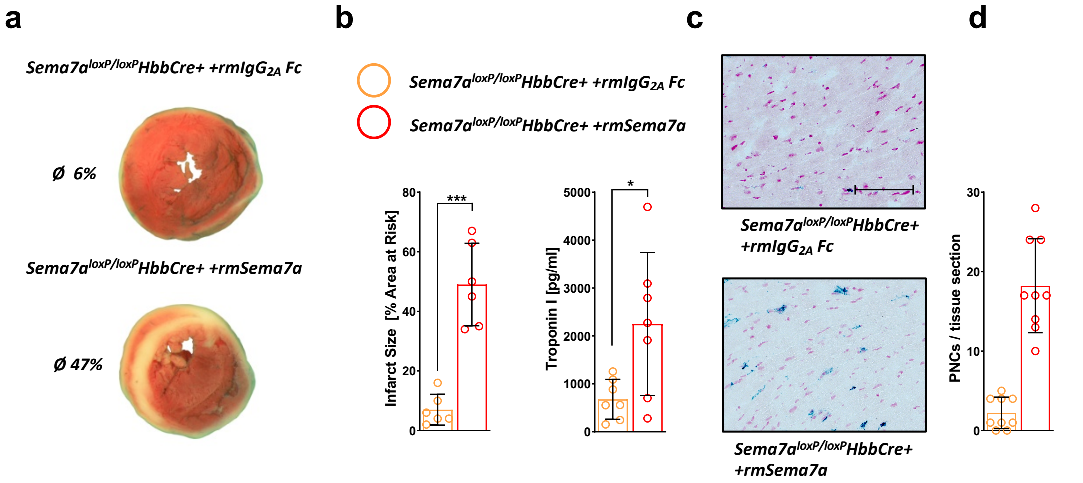

Supplementary Figure 11. Reconstitution of *Sema7a$^{loxP/loxP}$HBBCre+* results in increased MIRI. *Sema7a$^{loxP/loxP}$HBBCre+* animals or littermate controls were injected with either recombinant semaphorin 7a (rmSema7a) or Fc control (rmIgG$_{2A}$ Fc) and then subjected to 1 h of ischemia followed by 2 h reperfusion **a)** Representative TTC-stained slices of myocardial tissue showing infarcted area (blue/dark = retrograde Evans blue staining; red and white = AAR, white = infarcted tissue). **b)** Systematic evaluation of infarct sizes (% Area a Risk n=6/ group) and correlating troponin I plasma levels (n=7 /group) **c)** Representative histology sections (scale bar 100µm) stained for the presence of PNCs and **d)** number of PNCs counted from myocardial AAR sections in *Sema7a$^{loxP/loxP}$HBBCre+* animals injected with either recombinant semaphorin 7a (rmSema7a) or Fc control (rmIgG$_{2A}$ Fc) (n=9/ group). All comparisons in this figure were analyzed by unpaired two-tailed Student's *t*-tests (data are mean±SD; *p < 0.05 and ***p < 0.001 as indicated).

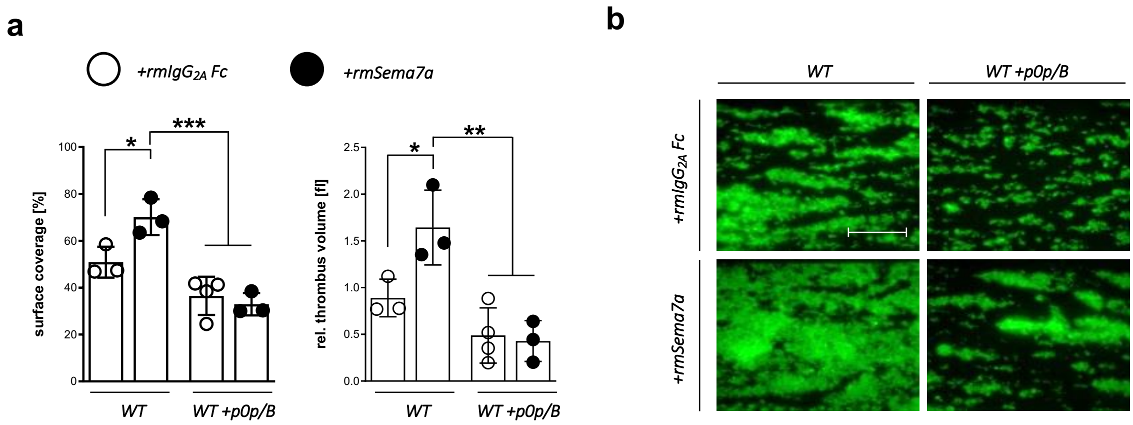

Supplementary Figure 12. Recombinant semaphorin 7A (rmSema7a) markedly enhances adhesion and thrombus formation of WT platelets on collagen under flow at a shear rate of 400 sec^{-1}. a) Blockade of the ligand-binding site of platelet GPIb (p0p/B) abolishes the thrombus-promoting effect of rmSema7a (Surface Coverage n=3;3;4;3). **b)** Representative fluorescence images (scale bar 50µm) as well as the mean surface coverage and relative thrombus volume are shown, as measured by integrated fluorescence intensity (IFI) per mm^2 (rel. thrombus formation n=3;3;4;3). For comparisons in this figure we performed one-way analyses of variance followed by Dunnett's tests to group WT+rmSema7a. (data are mean±SD; *$p < 0.05$, **$p < 0.01$ and ***$p < 0.001$ as indicated).

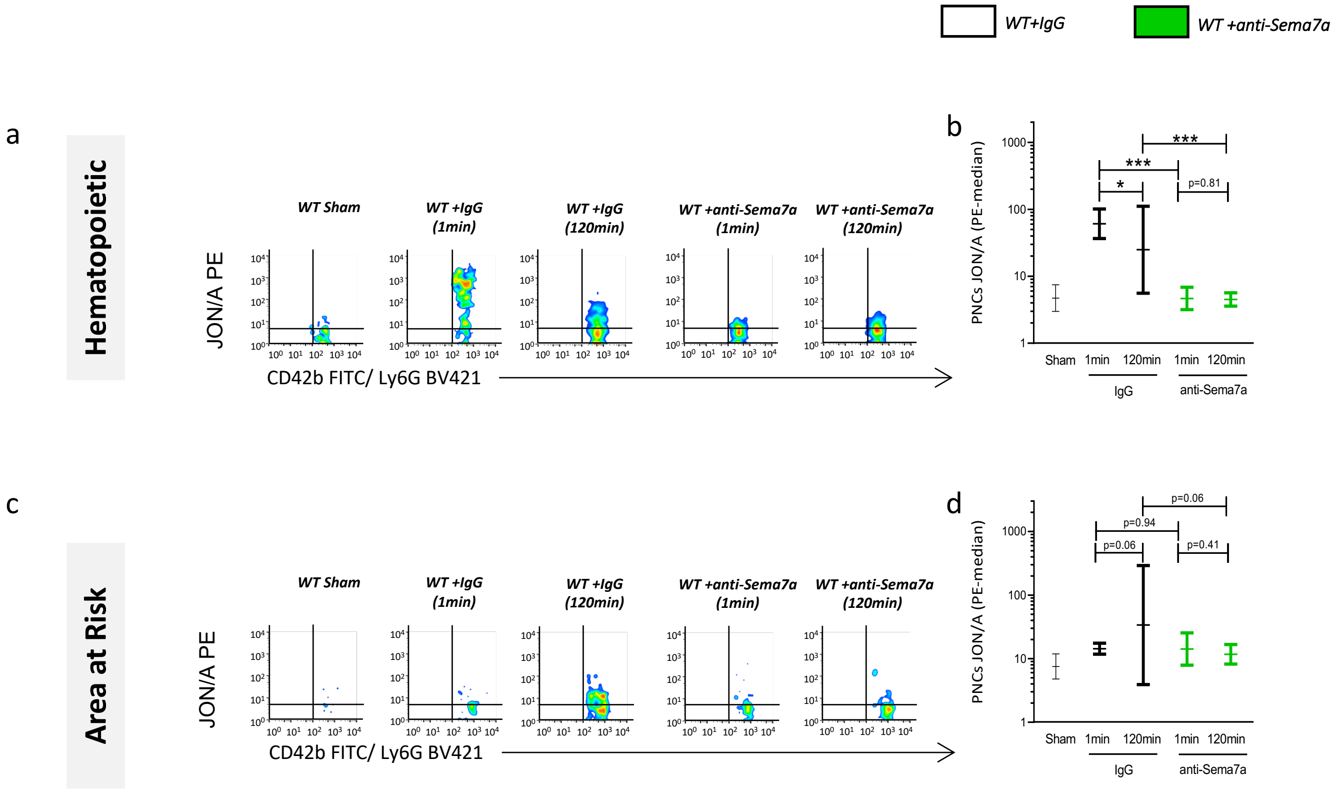

Supplementary Figure 13. Anti-Sema7a dampens integrin αIIbβ3 (JON/A) activation. Animals were subjected to 1 h myocardial ischemia and injected with either anti-semaphorin 7A antibody (anti-Sema7a) or IgG control 5 minutes before the reperfusion phase. Blood and myocardial tissue sections (AAR) were taken after 1 minute or 120 minutes of reperfusion. **a, b**) Representative flow-cytometric plots of PNCs in the blood and tissue of WT animals injected with either IgG control or anti-Sema7a showing integrin αIIbβ3 (JON/A) activation on PNCs after 1 min and 120 min reperfusion (n=5;4;3;4;5). **c, d**) Systematic evaluation of flow-cytometric mean fluorescence intensities of activated αIIbβ3 (JON/A) on PNCs of mice injected with anti-Sema7a or IgG control at 1 min and 120 min (n=4;5;3;4;4). For this figure we used log transformation of data to confirm normality. For log-transformed data, unpaired two-tailed Student's *t*-tests were performed on the log values and results are displayed as geometric means and their 95% confidence intervals (*p < 0.05 and ***p < 0.001).

Supplementary Methods

Murine myocardial ischemia and reperfusion model. Mice were anesthetized and placed on a temperature-controlled table to keep body temperature at 37°C. After intubation, ventilation was started, and parasternal thoracotomy was performed to expose the heart. An 8.0 nylon suture (Propylene, Ethicon, Norderstedt, Germany) was placed around the left coronary artery. Ischemia was induced for one hour by closing the suture and stopped for reperfusion. After the procedure, the heart was excised and blood plasma collected. Myocardium was cut into 0.8-mm-thick slices, and myocardial infarct size was ascertained by calculating the percentage of myocardial infarction compared to the area at risk (AAR) of each slice. The areas were identified using a double staining technique with Evans blue and triphenyltetrazolium chloride (TTC) [1]. The area at risk (AAR) and the infarct size were measured by planimetry using ImageJ 1.50i software. In subsets of experiments, blood was taken carefully by cardiac puncture, followed by harvesting the AAR after flushing the blood out after 1 minute or 120 minutes of reperfusion, and both were prepared for flow cytometry and protein measurements. Furthermore, hearts were excised after 120 minutes of reperfusion and transferred into Tissue-Tek® O.C.T.™ Compound (Sakura Finetek, Leiden, Netherlands) for cryopreservation and later histological staining.

Caspase 3 staining and Caspase 3 ELISA. Human cardiac myocytes (HCM-c, primary cell line, order-number: C-12810; lot-number: 9083205.4, PromoCell, Heidelberg, Germany) were grown to confluence on chamber slides followed by 6 hours of stimulation with rhSEMA7A, rhIgG$_1$ Fc, BSA or staurosporine (Sigma-Aldrich, Munich, Germany), all 1 µg/ml. After fixation, cells were stained with a 1:100 rabbit polyclonal anti-caspase3 antibody (abcam #ab44976 Cambridge, UK). After washing with staining buffer, cells were incubated with 1:500 goat anti-rabbit secondary polyclonal antibody conjugated with Alexa Fluor488 (Thermo Fisher

Scientific #A11008, Waltham, USA). Nuclei were counterstained with Roti-Mount FluorCare DAPI (Carl Roth, Karlsruhe, Germany). Imaging was processed on an Axiophot Zeiss microscope (Zeiss, Oberkochen, Germany) using a digital camera with Axio Vision software 4 .8. Human cardiac myocytes (HMC-c; PromoCell, Heidelberg, Germany) were grown to c onfluence in petri dishes for 1 week, followed by 1, 2, 4 or 24 hours of stimulation with rhSE MA7A, rhIgG$_1$ Fc (control; R&D SYSTEMS, Minneapolis, USA), BSA or staurosporine (posi tive control; Sigma-Aldrich, Munich, Germany), all 1 µg/ml. Cell lysates where then anal yzed by DuoSet® IC Human/Mouse Cleaved Caspase-3 (Asp175; #DYC835-2/DYC835-5) kit following the manufacturer's instructions.

Cardiac magnetic resonance imaging (MRI). Animals were subjected to cardiac MRI at 22 weeks of age. Anesthesia was induced with 3.5 vol.% isoflurane for 2 minutes. Mice were kept anesthetized with 1.0–2.5 vol.% isoflurane for a maximum of 30 minutes, and their body temperature was maintained with warm water blankets. To assess RV and LV mass and volumes, short-axis cardiac MRI images were acquired on a 70/39 7T BioSpec scanner (Brucker) using the IntraGateFLASH measuring method with the following parameters: echo time = 2.112 ms, 100 repetitions of 74.352 ms, 128-acquisition square matrix, spatial resolution of 176 µm. Ten axial slices of 1 mm thickness and 10 cardiac frames for reconstruction were taken to cover RV and LV.

Assessment of RV and LV -EDV, -ESV, -EF and mass by MRI. Analysis was performed on a clinical workstation with semi-automated contour-tracing software (CVI42, Release 4.1.8 (201), Circle Cardiovascular Imaging Inc., Calgary, Canada). We traced endocardial (for volume measurement) and epicardial contours (for wall mass defined as ventricular wall enclosed by endocardial and epicardial contours) of both ventricles. The quantitative analysis included two steps for both left and right ventricles: 1. tracing the endocardial contour in end-

diastolic and end-systolic phase for volume and function; 2. tracing the epicardial border in end-diastolic phase for wall mass. All measurements were performed on a minimum of five cardiac slices for each animal per condition.

Flow chamber experiments. Assembled chambers with 30 mm sandwiched glass capillaries (vitrotubes.com 0.02 mm path Length 0.20 mm width 0.014 mm wall) were cannulated with polyethylene tubing and fixed with epoxy glue. Capillaries were then coated for 2 h with 3.µg / ml E-selectin (R&D #724-ES) or with a mix of E-selectin and 3.5µg / ml ICAM1 (R&D #720-IC) and blocked with 1% casein (Sigma# C7594) for 1h at room temperature. Fresh human whole blood was mixed with 0,005 mg / ml rhodamine 6G (Sigma # 83697) and perfused to the capillaries, or incubated with rhSEMA7A (R&D #2068-S7-050) for 15 min at 37ºC before perfusion to the flow chambers. Videos are acquired on Hamamatsu ORCA-R^2 mounted on a Leitz microscope with a 200X magnification with a lens from Leitz Wetzlar 160/- EF L20/0.32 PHACO I and recorded and analyzed with Nikon NIS Elements Ar version 4.20 - 64bit (Nikon, Düsseldorf, Germany).

Flow-Cytometric Analysis. Blood was gently withdrawn from the heart left ventricle with a 25-G needle to a syringe coated 1:10 with citrate, and a sample of 100 µl was incubated in tubes previously warmed to 37°C with 1:100 Ab cocktail. Blood samples were stained at 37°C for 30 min in the dark and quickly lysed with warm 1x BD red blood cell lysis buffer (BD 555899), centrifuged at 300×g for 5 min at room temperature and fixed with 1x BD cell fix solution (BD 340181) for 10 min. After centrifugation at 300×g for 5 min at 4°C, samples were acquired in a BD FACSCanto II (BD-Heidelberg). For tissue analysis, the area at risk was collected, minced, and placed in 0.4 mg/ml collagenase (from *Clostridium histolyticum* - Sigma #C7657) that was diluted in 2% FBS with PBS for 30 min at 37°C for digestion. To inhibit the enzyme and wash the preparation, each sample was centrifuged at 400×g for 5 min at 4°C. After

discarding the supernatant, the cellular pellet was resuspended in 1 mg/ml collagenase-dispase solution (Roche #10269638001) and vortexed every 5 min for a total of 20 min and later washed with ice cold HBSS$^-$. After filtration, each heart sample was then concentrated and resuspended in 700 µl of 2% FBS in PBS$^-$. A total of 100 µl of heart sample was incubated with our flow cytometry antibody cocktail for 30 min on ice, washed with ice cold PBS$^-$ and fixed with 1x BD fixing buffer (BD 340181).

Intravital microscopy. Mice were anesthetized and cremastic tissues prepared under a Nikon 20x water dipping lens (NA=0,32). Neutrophils were labeled with 20 µl anti-Ly6G (biolegend #127608) and platelets with 20 µl of the platelet-specific FITC-labeled X488 antibody (EMFRET, Eibelstadt, Germany) resuspended in 200 µl and administered i.v.. Videos of postcapillary cremasteric venules of 20-40 µm in diameter were acquired on a Hamamatsu Orca Flash 4.0 camera mounted on a dual emission image splitter (optoSplit II, Cairn Research ; UK) with a rate of 16 frames/sec (on videos of 10sec total) and with a resolution of 2048 × 1 024 pixel mounted on a Nikon Eclipse Ci-L microscope (Nikon, Düsseldorf; Germany) run by NIS elements Ar software. Baseline videos were recorded before rmSema7a was administered i.v. and videos acquired after 15min of incubation for 5min in intervals of 20 sec. Neutrophil speed (µm /sec) was calculated from manually tracked cells, stationary neutrophils count (cell count / mm^2), platelet sedimentation on the vascular wall was measured (normalized MFI % / mm), transmigrated cells from the vasculature after 15min exposure to rmSema7a were count (cell count / mm^2), the distance of transmigrated cells measured in µm in all videos was processed by NIS elements Ar software 4.20 - 64bit (Nikon, Düsseldorf, Germany).

Supplementary References

1. Fishbein, M.C., *et al.* Early phase acute myocardial infarct size quantification: validation of the triphenyl tetrazolium chloride tissue enzyme staining technique. *Am Heart J* **101**, 593-600 (1981).
2. Granja, T., *et al.* Targeting CD39 Toward Activated Platelets Reduces Systemic Inflammation and Improves Survival in Sepsis: A Preclinical Pilot Study. *Crit Care Med* **47**, e420-e427 (2019).
3. Granja, T., *et al.* Using six-colour flow cytometry to analyse the activation and interaction of platelets and leukocytes--A new assay suitable for bench and bedside conditions. *Thromb Res* **136**, 786-796 (2015).
