# Supplementary material for: Red blood cell-derived semaphorin 7A promotes thrombo-inflammation in myocardial ischemia-reperfusion injury through platelet GPIb
Source: Nat Commun. 2020 Mar 11;11:1315. doi: 10.1038/s41467-020-14958-x (PMC7066172; doi:10.1038/s41467-020-14958-x)
Supplement: Supplementary file 3 — Description of Additional Supplementary Files [file 41467_2020_14958_MOESM3_ESM.docx]

**Supplementary Movie 1. Sema7A influences neutrophil rolling and migration in IVM.** Representative optosplit videos concentrated on the 15min time point of platelets (cyan) and neutrophils (magenta) travelling on mouse cremaster micro-vasculature (≤40 µm) with references how data is; white cross represents count stationary cells; white lines connect (µm) transmigrated neutrophils to vasculature exit points; red lines delineate the intensity profile of platelets attachment to vasculature as mean fluorescence intensity (MFI); Green circles track moving neutrophils across a defined region which is translated into cell path speed (µm / sec). Intravital microscopy experiments were performed on n=4 mice with n=10 videos of untreated and after i.v. inoculation with rmSema7a treated groups. From IVM data analysis we calculated the neutrophil speed (µm / sec) was calculated from tracked cells; stationary neutrophils count (cell count / mm^2^); platelet sedimentation on the vascular wall was measured (normalized MFI % /mm); transmigrated cells from the vasculature after 15min exposure to rmSema7a were count (cell count / mm^2^); the distance of transmigrated cells measured in µm. All analyzed data is plotted in Supplementary Figure 6.
