# Supplementary material for: Red blood cell-derived semaphorin 7A promotes thrombo-inflammation in myocardial ischemia-reperfusion injury through platelet GPIb
Source: Nat Commun. 2020 Mar 11;11:1315. doi: 10.1038/s41467-020-14958-x (PMC7066172; doi:10.1038/s41467-020-14958-x)
Supplement: Supplementary file 5 — Reporting Summary [file 41467_2020_14958_MOESM5_ESM.pdf]

nature research

Corresponding author(s): Peter Rosenberger

Last updated by author(s): Feb 5, 2020

Reporting Summary

Nature Research wishes to improve the reproducibility of the work that we publish. This form provides structure for consistency and transparency in reporting. For further information on Nature Research policies, see Authors & Referees and the Editorial Policy Checklist .

Statistics

For all statistical analyses, confirm that the following items are present in the figure legend, table legend, main text, or Methods section.

n/a	Confirmed	
☐	☒	The exact sample size (*n*) for each experimental group/condition, given as a discrete number and unit of measurement
☐	☒	A statement on whether measurements were taken from distinct samples or whether the same sample was measured repeatedly
☐	☒	The statistical test(s) used AND whether they are one- or two-sided<br>*Only common tests should be described solely by name; describe more complex techniques in the Methods section.*
☒	☐	A description of all covariates tested
☐	☒	A description of any assumptions or corrections, such as tests of normality and adjustment for multiple comparisons
☐	☒	A full description of the statistical parameters including central tendency (e.g. means) or other basic estimates (e.g. regression coefficient) AND variation (e.g. standard deviation) or associated estimates of uncertainty (e.g. confidence intervals)
☐	☒	For null hypothesis testing, the test statistic (e.g. *F*, *t*, *r*) with confidence intervals, effect sizes, degrees of freedom and *P* value noted<br>*Give P values as exact values whenever suitable.*
☒	☐	For Bayesian analysis, information on the choice of priors and Markov chain Monte Carlo settings
☒	☐	For hierarchical and complex designs, identification of the appropriate level for tests and full reporting of outcomes
☒	☐	Estimates of effect sizes (e.g. Cohen's *d*, Pearson's *r*), indicating how they were calculated

Our web collection on statistics for biologists contains articles on many of the points above.

Software and code

Policy information about availability of computer code

Data collection	FACS Diva version 6.0<br>NIS Elements Ar version 4.20
Data analysis	FlowJo 10.0<br>GraphPadPrism 8.1.2<br>JMP 14.2.0<br>ImageJ 1.50i

For manuscripts utilizing custom algorithms or software that are central to the research but not yet described in published literature, software must be made available to editors/reviewers. We strongly encourage code deposition in a community repository (e.g. GitHub). See the Nature Research guidelines for submitting code & software for further information.

Data

Policy information about availability of data

All manuscripts must include a data availability statement. This statement should provide the following information, where applicable:

- Accession codes, unique identifiers, or web links for publicly available datasets
- A list of figures that have associated raw data
- A description of any restrictions on data availability

Data are provided in source file

Field-specific reporting

Please select the one below that is the best fit for your research. If you are not sure, read the appropriate sections before making your selection.

[x] Life sciences [ ] Behavioural & social sciences [ ] Ecological, evolutionary & environmental sciences

For a reference copy of the document with all sections, see nature.com/documents/nr-reporting-summary-flat.pdf

Life sciences study design

All studies must disclose on these points even when the disclosure is negative.

Sample size

Determined statistical analysis with the department of biometrics UKT

Data exclusions

Throughout this study all data was tested for normality. Normal distribution was checked using skewness tests. Small data sets that may lead to wrong interpretations were accessed visually by inspection of the data distribution using statistically relevant histograms. Whenever data inspection revealed skewed distribution of results, data log-transformation was performed to achieve normality.

Replication

Achieve minimal number of n=5 specimens per group

Randomization

Randomization was achieved on specimen groups while filling experiments according to the availability of the animal specimens not following any specific order

Blinding

Not applicable

Reporting for specific materials, systems and methods

We require information from authors about some types of materials, experimental systems and methods used in many studies. Here, indicate whether each material, system or method listed is relevant to your study. If you are not sure if a list item applies to your research, read the appropriate section before selecting a response.

Materials & experimental systems

n/a	Involved in the study	
[ ]	[x]	Antibodies
[x]	[ ]	Eukaryotic cell lines
[x]	[ ]	Palaeontology
[ ]	[x]	Animals and other organisms
[ ]	[x]	Human research participants
[x]	[ ]	Clinical data

Methods

n/a	Involved in the study	
[x]	[ ]	ChIP-seq
[ ]	[x]	Flow cytometry
[x]	[ ]	MRI-based neuroimaging

Antibodies

Antibodies used

rat anti-mouse Ly6G (Biolegend# 127628, clone 1A8) labeled BV421
rat anti-mouse CD42b-FITC (Emfret# M040-1, clone Xia.G5) labeled FITC
rat anti-mouse CD62P (BD Pharmingen# 563674, clone RB40.34) labeled Alexa Fluor 647
rat anti-mouse activated GPIIb/IIIa (Emfret# M023-2 , clone JON/A,) labeled PE

Validation

FACS antibody validation described here: Granja T, Schad J, Schussel P, Fischer C, Haberle H, Rosenberger P, Straub A. Using six-colour flow cytometry to analyse the activation and interaction of platelets and leukocytes - A new assay suitable for bench and bedside conditions. Thromb Res 2015; 136: 786-796.

Animals and other organisms

Policy information about studies involving animals; ARRIVE guidelines recommended for reporting animal research

Laboratory animals

All experimental animals were sex randomized and C57BL/6 background and all protocols performed with specimens weighing 25g and with at least 6 weeks old. Sema7a-/- mice are described here: Pasterkamp, R.J., Peschon, J.J., Spriggs, M.K. & Kolodkin, A.L. Semaphorin 7A promotes axon outgrowth through integrins and MAPKs. Nature 424, 398-405 (2003). Sema7a loxP/loxP mouse line was acquired from Ozgene and cross-bread with available Cre recombinase–positive mouse line erythrocyte specific HbbCre+, myocardial cell specific Myh6Cre+, endothelial cell specific Tie2Cre+ and neutrophil specific LysMCre+.

Wild animals

Not Applicable

Field-collected samples	Not Applicable
Ethics oversight	Animal experiments followed all German guidelines for use of living animals laboratory applications and were approved by the Institutional Animal Care and the Regierungspräsidium Tübingen and Würzburg, and the Landesamt für Verbraucherschutz Niedersachsen.

Note that full information on the approval of the study protocol must also be provided in the manuscript.

Human research participants

Policy information about studies involving human research participants

Population characteristics	During coronary intervention, all blood samples were collected at the end of cardiopulmonary bypass or during occlusion of the coronary arteries during off pump cardiac surgery. For another set of experiments samples were collected from 22 human subjects of randomized age and sex and analyzed for their native content of Semaphorin7A.
Recruitment	Written informed consent was obtained from each patient before samples were taken. Patient samples before and after cardiac surgery were collected as part of the TüSep-Study (NCT02692118).
Ethics oversight	All experiments carried on human samples were consented by formal approval handed by the ethics committee (Institutional Review Board) of University of Tübingen. Samples of patients with myocardial infarction were obtained at presentation to the catheter laboratory and processed (Biobank: 266/2018BO1; Sema7a subanalysis: 266/2018BO2; Clinicaltrial.gov: NCT01417884).

Note that full information on the approval of the study protocol must also be provided in the manuscript.

Flow Cytometry

Plots

Confirm that:

- [x] The axis labels state the marker and fluorochrome used (e.g. CD4-FITC).
- [x] The axis scales are clearly visible. Include numbers along axes only for bottom left plot of group (a 'group' is an analysis of identical markers).
- [x] All plots are contour plots with outliers or pseudocolor plots.
- [x] A numerical value for number of cells or percentage (with statistics) is provided.

Methodology

Sample preparation	Whole blood preparation: blood was gently withdrawn from the heart left ventricle with a 25-G needle to a syringe coated 1:10 with citrate, and a sample of 100 µl was incubated in tubes previously warmed to 37°C with 1:100 Ab cocktail. Blood samples were stained at 37°C for 30 min in the dark and quickly lysed with warm 1x BD red blood cell lysis buffer (BD 555899), centrifuged at 300×g for 5 min at room temperature and fixed with 1x BD cell fix solution (BD 340181) for 10 min. After centrifugation at 300×g for 5 min at 4°C all cells were resuspended in 500µl and acquired in a FACS CantoII.<br><br>For tissue analysis: the area at risk was collected, minced, and placed in 0.4 mg/ml collagenase (from Clostridium histolyticum - Sigma #C7657) that was diluted in 2% FBS with PBS for 30 min at 37°C for digestion. To inhibit the enzyme and wash the preparation, each sample was centrifuged at 400×g for 5 min at 4°C. After discarding the supernatant, the cellular pellet was resuspended in 1 mg/ml collagenase-dispase solution (Roche #10269638001) and vortexed every 5 min for a total of 20 min and later washed with ice cold HBSS-/-. After filtration, each heart sample was then concentrated and resuspended in 700 µl of 2% FBS in PBS-/-. A total of 100 µl of heart suspension was incubated with our flow cytometry antibody cocktail for 30 min on ice, washed with ice cold PBS-/- and fixed with 1x BD fixing buffer (BD 340181). After centrifugation at 300×g for 5 min at 4°C all cells were resuspended in 500µl and acquired in a FACS CantoII.
Instrument	BD FACSCanto II (BD-Heidelberg) - serial number is missing
Software	BD FACSDiva software (Version 6, BD, Heidelberg, Germany)
Cell population abundance	In this work there is no cell sorting.<br>Whole blood samples were acquisition was stopped when 10000 events were reached.<br>On heart area at risk samples, the number of infiltrated neutrophils was accessed after the acquisition of 50000 cells
Gating strategy	In both whole blood samples and processed heart area at risk, sample acquisition was focused on peripheral granulocytes by their granularity and surface expression of lymphocyte antigen 6 complex, locus G (Ly-6G), noted as SSC/Ly-6G+. The presence of platelet surface marker CD42b on the surface of SSC/Ly-6G+ events distinguished platelet-neutrophil complexes SSC/Ly-6G+/CD42b+ (PNCs) from free circulating PMNs SSC/Ly-6G+/CD42b-. These two populations were tested for their display of surface transmembrane glycoproteins P-selectin (CD62P) and activated GPIIb/IIIa (clone JON/A)

- [x] Tick this box to confirm that a figure exemplifying the gating strategy is provided in the Supplementary Information.
